# Assessing motor development with wearables in low-resource settings: feasibility in rural Malawi

**DOI:** 10.1038/s41390-025-03818-3

**Published:** 2025-03-03

**Authors:** Elisa Taylor, Manu Airaksinen, Rikhard Ihamuotila, Milja Kivelä, Ulla Ashorn, Leena M. Haataja, Charles Mangani, Sampsa Vanhatalo

**Affiliations:** 1 BABA Center, Pediatric Research Center, Department of Clinical Neurophysiology, New Children’s Hospital and HUS Imaging, Helsinki University Hospital, Helsinki, Finland; 2 Department of Physiology, University of Helsinki, Helsinki, Finland; 3 Center for Child, Adolescent and Maternal Health Research, Faculty of Medicine and Health Technology, Tampere University and Tampere University Hospital, Tampere, Finland; 4 Research Unit of Clinical Medicine, MRC Oulu, Oulu University Hospital and University of Oulu, Oulu, Finland; 5 Department of Pediatric Neurology, Children’s Hospital, Helsinki University Hospital and University of Helsinki, Helsinki, Finland; 6 Kamuzu University of Health Sciences, Blantyre, Malawi

## Abstract

**Background:**

Tracking of early motor development is essential for all neurodevelopmental assessments. A multisensor wearable system, MAIJU (Motor Assessment of Infants with a JUmpsuit), was recently developed for an objective and scalable measurement of developing motor skills in out-of-hospital settings. Here, we assessed its feasibility in remote low-resource settings.

**Methods:**

We recruited 44 infants for repeated at-home measurements (total *N* = 121) with the MAIJU wearable in rural Malawi. We assessed (i) technical quality of the measured wearable data, (ii) reliability of the cloud-based analysis outputs, and (iii) maternal user experience. A dataset from 47 infants (total *N* = 111 measurements) in Finland served as a reference from a high-resource environment.

**Results:**

Altogether 94% of the measurements were technically successful. The analysis outputs from the automated cloud pipeline were all comparable to the reference cohort in Finland. The method was rapidly learned by the local study personnel, and it was well received by the mothers.

**Conclusion:**

Our results suggest that advanced multisensor wearables and cloud-based analytics can be readily used in remote and low-resource settings. Uptake of such objective methods holds promise for harmonizing and increasing equality in developmental assessments, as well as facilitating a wide range of global health studies on early life.

**Impact:**

- Motor development is an effective measure of infants’ overall neurodevelopment.^1–4^ A multisensor wearable system was recently developed for an objective and scalable tracking of infants’ developing gross motor skills.^5–7^ Here, we assessed feasibility of using such wearable systems in low-resource settings in rural Malawi. Our findings show that the measurements are technically reliable, the outputs from the cloud-based analysis pipeline are comparable to those from our reference study in Finland, and the wearable recordings are well-received by the parents. The findings support the use of multisensor wearables in remote settings and highlight their potential for benchmarking early-life global health studies.

## Introduction

Objective evaluation of early motor performance is essential for wider neurodevelopmental assessment in all health care, developmental research, as well as for benchmarking novel therapeutic interventions. Currently, robust early tracking of motor development is challenged by the lack of objective measures and a wide individual variation. Also, the existing methods are sensitive to both the testing environment and the assessor’s expertise.^8–12^

The recent progress in wearable technology and its associated, machine-learning based signal analyses have enabled reliable and objective assessment of infants‘ developing motor performance in out-of-hospital settings.^5,6^ Our recent studies have developed and validated a multisensor wearable system and its deep learning-based analytic pipeline, collectively named MAIJU system (Motor Assessment of Infants with a JUmpsuit),^7^ which was shown to provide an accurate quantification and developmental tracking of infants motor performance using at-home measurements in a high-resource setting in Finland.

In the global health context, there is a wide need for scalable, reliable, and affordable methods that could be used to track early life development.^13^ Here, we assess whether an advanced wearable system, such as MAIJU, could be used reliably in geographically remote low-resource settings. We conducted a feasibility study as the first step in our prospective project on mapping early motor development in infants in rural Malawi. This study aimed to assess (i) the technical quality of the recordings performed by the local data collectors in such environments, and (ii) the performance and reliability of the fully automated, cloud-based analysis pipeline used directly from the study site, and (iii) the reception, or user experience of the mothers (Supplementary file 4) when wearable recordings are performed in their homes.

## Methods

### Study overview

**Fig. 1:**
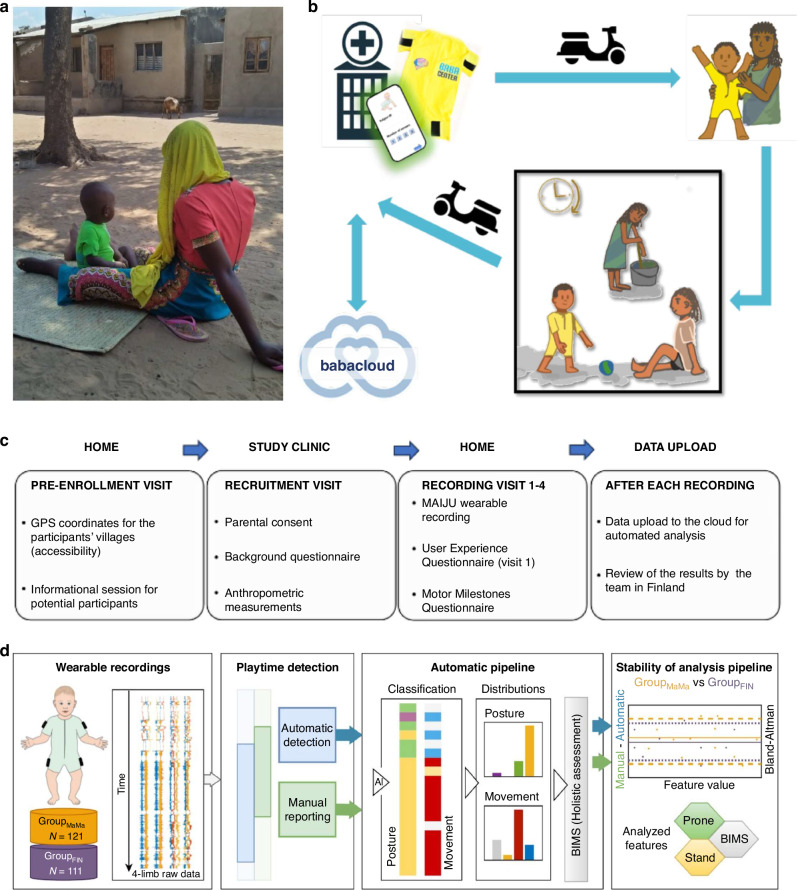
Study overview. **a** Recording session, **b** MAIJU protocol: the data collector visits the participant’s home to perform the recording; the data is uploaded to the cloud system for an automated analysis after recording session, **c** Feasibility study design, **d** Overview of the data analysis.

An overview of the study is shown in Fig. 1. This feasibility study is the first part of a wider study that aims to characterize early motor development with wearable recordings as a reference in future studies. Recruited infants had repeated at-home measurements by study data collectors using MAIJU wearable, and the data was automatically analyzed using a cloud-based analysis platform. This feasibility study aimed to characterize possible challenges with wearable recordings in rural areas. Specifically, we qualitatively evaluated the study logistics, assessed technical data quality, compared the outputs of analytic pipelines to a parallel cohort study in Finland, and surveyed maternal experience. The full MaMa study was approved by the regulatory authorities in Malawi (COMREC P.11/22/3875/Mangani 25. May 2023; Clinicaltrials.gov #NCT057826733).

### Study participants

The study population comprises 44 participants recruited between 4.6 and 8.5 months of age from villages in rural Malawi, specifically within the Lungwena area in Mangochi district in southern Malawi. Exclusion criteria included suspected preterm birth (at seventh month or earlier) based on maternal information, apparent congenital malformation or severe illness, or visual impairment, as clinically determined by a study nurse.

### Recruitment and study visits

#### Recruitment

Prior to recruitment of the study participants, the local data collectors were trained to perform MAIJU recordings. The training was carried out by the study team from Finland during a two-week visit to the study site. Further training was provided remotely at a later stage when required. All training was delivered in English with both the training staff and trainees having professional proficiency in the language. Infants were recruited to the feasibility phase of the study during June 2023 to October 2023, including all eligible and consenting participants. The potential villages were selected based on their proximity to Lungwena Health Center. The villages were first visited to inform about the study and collect GPS coordinates for the purpose of evaluating the accessibility of the village. Study personnel and village chiefs convened meetings where all mothers with recruitment-age infants were gathered for informational sessions. During these meetings, names, dates of birth, and addresses were documented. Subsequently, data collectors identified eligible participants, specifically those nearing the age of five months. These mother-child dyads were registered using the data collection software, CommCare, and invited to the health center for enrollment, during which the informed consent was obtained using participants’ native language Chiyao and Chichewa together with anthropometric measurements and background information (i.e., mother’s socioeconomic situation and obstetric history). All participation was voluntary, and a signed or thumb-printed informed consent was obtained from the infants’ caregiver.

#### Study visits

Post-enrollment visits took place at infants’ homes where MAIJU recordings were performed as supervised recordings every 6-8 weeks by trained data collectors. The recordings were conducted under supervision, as delivering the wearable to the participants’ homes was not feasible; most houses in the rural villages do not have an address, only a GPS location, and sometimes, the house can only be located by asking locals. Also, as many Malawian mothers have limited reading ability, it was practical for the supervising data collectors to give the instructions for each recording session. Prior to a visit, the families were informed by the data collectors of the specific day scheduled for the recording. On the day of the visit, the data collector commuted to the household using either a motorcycle or a mountain bike, occasionally opting to walk or utilize a project car in case of technical issues with the bikes.

### MAIJU wearable system

The MAIJU wearable system (Fig. 1b) consists of a whole-body garment fitted with four waterproof movement sensors, a mobile device running a custom-made iOS application “Maijulogger” (Kaasa GmbH, Düsseldorf, Germany), and a fully automated cloud-based analytic pipeline, “Babacloud” (www.babacloud.fi).^7^ In the present MAIJU version, the movement sensors transmit synchronized data (sampling rate 52 Hz, tri-axial, both accelerometer and gyroscope) to a mobile device using a low-energy Bluetooth (BLE) connection. The data was initially saved in the mobile device, and it was uploaded via mobile networks to the cloud server after the recording session to be processed with the fully automated analysis pipelines.

### MAIJU recording session

Before each recording session the data collector re-explained the study. The mother was advised to let the child roam freely and to avoid unnecessary carrying or physical support unless needed by the infant for nursing, safeguarding, or soothing to maximize the infant’s natural motility in the movements. If needed, a colorful ball was given to the infant to encourage spontaneous, free play. At each recording session (Fig. 1a, b), the data collector started data recording in the Maijulogger application and placed the sensors in the correct order and orientation into the suit pockets.^7^ Then, the suit was dressed on the infant after ensuring that the infant was fed, wearing a diaper, and feeling well. If the child was seemingly sick or unwell, the recording was canceled, and the child was referred to the nearest health center. During the measurement, the infant was encouraged to play and move around freely (‘free playtime’), and there was no limitation to moving between rooms or into or out of the house. The data collector stayed at sight distance from the child to avoid interfering with the child’s activity, while also making general notes about events that could help in later interpretation of the results (e.g. times of eating, sleeping, carrying, playing).

Each MAIJU recording session was instructed to continue for the duration necessary to accumulate at least a total of one hour of free play, which could be accumulated from several shorter epochs. The data collector defined free play as infant playing or moving independently without physical assistance.

During each session, the data collectors also filled an electronic Case Report Form (eCRF) that was hosted in the safe networked setting offered by CommCare. During the recording, the data collector listed epochs of free play, feeding, and sleeping schedules, as well as other unexpected events that may have affected the recording. These data were collected to ensure data integrity and to support any possible needs for technical troubleshooting afterwards. Notably, the default analysis pipeline processed data fully automatically, without using manual annotations. Other information recorded to the electronic form by the data collector included: observations about infants’ reaching of motor milestones, child’s health information, and general information about infants’ living environment (e.g., parents’ occupation, family composition, tribe, language). During the first visit, the data collector also interviewed the mother for a separate User Experience Questionnaire (Supplementary file 4), or in practice, the mothers’ perception about using MAIJU wearable for studying their infants at home.

### Maternal user experience

Maternal views (user experience, Supplementary file 4) about MAIJU wearable recordings were assessed during the first study visit by completing a questionnaire with the following open format questions and a numerical rating: (i) were there challenges with the MAIJU wearable or did it make markings on the skin? (ii) how did the parents and the child feel about the wearable? (iii) did the MAIJU recording affect the child’s behavior in any way?, and (iv) how did they feel about MAIJU wearable on a scale of 0 to 3 (0 = very bad, 1 = reasonable, 2 = good, 3 = excellent). It could have been interesting to also have parental feedback on the study reasons or the overarching rationale, however such was not feasible because the study plan had to be fixed and approved by the national ethics board before approaching the parents.

### Data analyses

#### Overview

The results were analyzed at two levels: (i) a general evaluation of the study protocol and related logistics, and (ii) a technical and computational evaluation of the measurement data. For benchmarking the present findings (Group_MaMa_), we used a dataset from an ongoing study in Helsinki, Finland (Group_FIN_).

#### Benchmarking with the dataset recorded in Finland

When relevant, we compared the present observations to our prior experience and findings from a largely comparable longitudinal study that is ongoing in Helsinki, Finland.^5,6^ There were some logistical differences between the studies in Malawi and Finland; in Malawi, MAIJU recordings were performed by trained data collectors (supervised), whereas in Finland, recording sessions were conducted by parents (unsupervised), with the suit delivered to homes via courier service. When carried out as supervised recordings, the recording sessions were shorter as the study data collector was able to observe in real time, and end the recording, when sufficient length of spontaneous play activity had been collected.

#### Criteria of technical recording success

Each recording session was defined technically successful when (i) at least 30 minutes of collected data was obtained from at least three of four sensors, (ii) data upload to Babacloud from the mobile device was successful, and iii) data analysis with the fully automated computational pipeline was successful.

#### Technical assessment of recordings using the automated analysis pipeline

As described in detail before, the automated analysis pipeline in the computational Babacloud provides a two-phase analysis.^5–7^ First, there is an automated detection of the recording segments with free playtime (spontaneous activity) and carrying of the infant, which are used to identify epochs for subsequent motor quantitation. The free playtime classifier has been trained on parental reports deriving from the Finnish reference dataset. Second, there is a second-to-second level detection of infant’s posture and movement types; they are used directly to generate aggregated measures of motor performance, such as computing times spent in each posture or assessing the motor maturity with a holistic index like BIMS (*BIMS; Baba Infant Motor Score*).^5,7^

In this feasibility study, we wanted to analyze the overall stability and replicability of the automated analysis pipeline. Initially, we compared the analysis outputs for the full range of different motor performances obtained after two preprocessing options: automatically detected playtime vs. playtimes noted in the diaries of the study data collectors. The recording sessions in Malawi were performed by a data collector who was observing and taking notes about the child’s activity to ensure that sufficient free playtime was collected during each measurement. In the reference dataset in Finland, the notes about the child’s free playtime during the recording at home were made by the child’s parents.

Further, we used Bland-Altman (B-A) analyses, where selected quantified motor measures are compared between the automated (algorithmic) and the manual (observed by the data collector) playtime detections. We reasoned that B-A analysis of this kind will indicate any bias across the range of motor skills. Moreover, distributions of the difference (error in the B-A plot) could be directly compared between the MaMa cohort and the reference cohort from Finland. We hypothesized that these distributions are not significantly different between Group_MaMa_ and Group_FIN_ cohorts if the automated pipeline (trained on the Finnish cohort) generalizes to the measurements and the population in Malawi. Notably, this assessment only focuses on the generalizability of the analysis pipeline, and it does not aim to study potential differences in early motor development between countries. Scatter plots were used to visualize the relationship between the automated and manual playtime detections.

### Statistical analyses

Group comparisons were examined using non-parametric Mann-Whitney U test (not normal distribution) or parametric two-sample independent t-test (normal distribution). Ratios of within groups were compared using the chi-square test for independence. The effect size was calculated with Cohen’s d. All statistical tests considered a significance level of α = 0.05.

## Results

### Characteristics of the study cohort

Between June and October 2023, a total of 44 infants (22 males and 22 females) were enrolled in the feasibility phase of the MaMa study (Group_MaMa_), and 1-4 MAIJU wearable recordings per child were performed at 6–8 week interval. This yielded a total of 121 recordings. The age at recording ranged from 5.0 to 10.0 months (mean age 7.4 ± 1.2 months (SD)). The benchmark dataset from Finland (Group_FIN_) comprised 47 participants (25 males and 22 females). There were no significant differences in age distribution and sex ratio between Group_MaMa_ and Group_FIN_ (Supplementary file 1). Regarding other demographic and social backgrounds, the participants came mainly from the Yao and Chewa populations. The house size of the families ranged from one to three rooms. Approximately one-third of the mothers and two-thirds of the fathers were able to read. The main occupations were farming (mothers) and fishing (fathers). For further details (Supplementary file 2).

### Technical quality of the wearable recordings

The overall technical success rate was 94% (n = 121) for the MAIJU recordings (>30 min data and a successful data analysis via cloud pipeline). During the early phase of the feasibility study, there was a transient technical challenge related to the Bluetooth connections of a specific iOS device model, which was solved by changing to new iOS models.

We then closely inspected the seven (6%) recordings with technical failures. They were from six different infants, so the failure was not related to the home or recording environment. The failed recordings occurred within the first two months of the feasibility study (failed recordings on study days 8-58 vs. successful recordings on study days 1-135 (U = 166.000, Z = −2.587, *p* < 0.010) suggesting that they could relate to less experience by the data collectors. The early phase of the study was also reflected in the younger age of the infants in the failed recordings (U = 189.500, Z = −2.381, *p* < 0.017, Supplementary File 3). Several other factors showed no difference between failed and successful recordings: the ID of the study assistant, wall material at home, recording surface (sand, sand/mud indoors, concrete), total eating time, and total sleeping time.

Additional recording notes indicated child-related reasons for five of seven technically compromised recordings; two recordings were stopped after the child was found to be seemingly unwell (sick) during the recording, while three recordings were stopped due to excessive crying of the child, which prevented spontaneous movement activity. One recording was interrupted due to a datalogger crash, which was considered clearly technical in nature.

Later interviews of the data collectors revealed that they felt somewhat uncomfortable staying at participants’ homes for several hours in the beginning of the study, which impacted the duration of the first recording sessions. Further training of the study assistants improved the length of the recording sessions and tackled the technical issues (i.e., lost connection, data logger crash) during the rest of the feasibility study.

### Assessment of the automated analysis pipeline

**Fig. 2:**
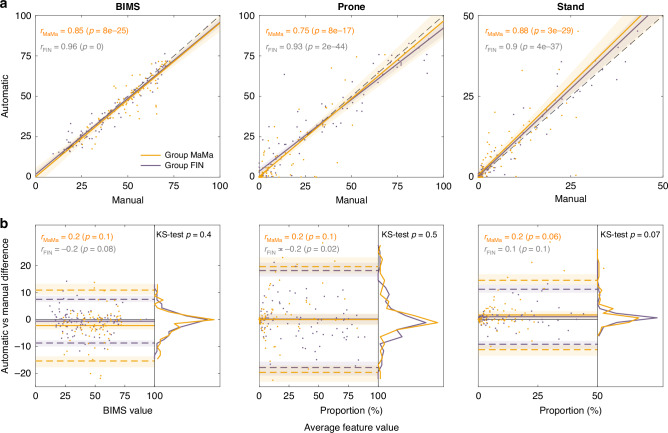
Performance of the automated analysis pipeline. The example scatter plots (**a**) and Bland-Altman plots (**b**) show the correspondence between results obtained after manual (human observer) vs. automated (AI algorithm) playtime detections. The findings are shown for the holistic motor measures (BIMS, scale 0-100) and the time spent in prone or standing postures. Note their strong correlation (scatter plots), lack of significant bias as a function of motor measure (**b**), as well as the highly comparable distributions (KS-test) of data from Malawi (orange) and Finland (gray).

The performance of the analysis pipeline was assessed holistically by comparing quantified motor measures between the automated and the manual playtime detections (Fig. 2). The scatter plots in Fig. 2a show a strong concordance between motor measures obtained after automated vs. manual playtime detections, and very concordant findings in both Group_MaMa_ and Group_FIN_. Further assessment for systematic errors with the B-A plots (Fig. 2b) compared infants’ time spent in prone or standing postures as well as a holistic assessment of infants’ motor maturity using their BIMS score.^5,7^ The outputs are very comparable across the full range of motor performances between the automated and manual playtime detections, and there was no difference in the B-A distributions between Group_MaMa_ and Group_FIN_. Together, these indicate that the outputs of the full analysis pipeline are stable and reproducible in the cohort from Malawi.

### Practical experience, home visits for the recordings

#### Commute

Roads to the households varied, ranging from tarmac roads with frequent potholes to dirt roads and narrow paths. Accessibility was occasionally hindered during heavy rains in the rainy season. It was not related to the MAIJU method per se, but it could have resulted in re-scheduling of appointments.

#### Recording session

The MAIJU recordings were typically performed with the infant playing on the porch or under a central tree in the village, or inside the house in case of rain. The length of the recording session was not standardized as the spontaneous playtime epochs during each session are often unpredictable. The aim was to record at least a total of 1 hour of the infant’s spontaneous movement (‘play time’), which was achieved with recording sessions lasting for 1.5-2 hours.

### Maternal user experience

Altogether 41 (93%) mothers provided responses to the User Experience Questionnaire (Supplementary file 4). The MAIJU wearable was seen favorably by all mothers, with the mean score between good and excellent (mean 2.34 ± 0.480). Specifically, 65.9% of parents described the device as ‘good’, while 34.1% rated it as ‘excellent’. None of the mothers rated the MAIJU experience as ‘very bad’ or ‘reasonable’. None of the mothers reported any challenges or skin markings from the use of the MAIJU wearable. There were no reports of the suit feeling too hot, even when temperatures occasionally rose above 30°C. The verbal responses included one comment describing the suit as ‘just like other locally bought clothing’. Two parents mentioned that their child felt ‘strange wearing the wearable’, although they still rated their feeling as ‘good’ in the numerical grade.

## Discussion

Our results show that at-home assessment of infants’ motor performance can be performed effectively and reliably using the advanced MAIJU wearable system in a remote, low-resource setting. No persisting or significant impediments were encountered with the practical implementation, technical performance, or using the cloud-based automated analysis pipeline. Regarding practical feasibility, this work compares well with the results of prior studies reporting the use of conventional actigraphy in low-resource settings.^14,15^ Our work extends all prior studies by showing that it is also possible to use an advanced multi-sensor wearable system to assess and track infant’s motor performance at high accuracy and quantitative detail, far beyond the traditional activity counts provided by the wrist or ankle worn actigraphy.

Objective and reliable assessment of infants’ developmental performance is essential for benchmarking a large range of developmental studies, including measuring efficacy in interventions. Several methods have been introduced for early neurodevelopmental assessments in the low-resource and/or out-of-hospital settings. They are typically based on combining maternal questionnaires and/or direct infant observations to provide a multidimensional assessment,^16–18^ with at best reasonable reliability, but only in trained hands. In addition, novel technologies have been attempted, including infants’ eye tracking, to assess early cognitive development - however, these methods are challenged by the need for expertise and the often-suboptimal sensitivity, replicability, and generalizability.^19–21^ As a complement to these primarily non-motor assessments, the MAIJU wearable system was recently developed for quantifying early motor performance and abilities in a natural out-of-hospital setting.^5–7^ It was shown to support a reliable longitudinal tracking of early motor development in larger infant cohorts in Finland,^5,6^ where measurements are performed at homes by the parents (i.e., unsupervised). Our present feasibility study shows that the MAIJU wearable method can be taken up rapidly by a novel study team even in very different environments, such as rural Malawi. Benchmarking with the ongoing studies in Finland proved comparable technical success in nearly all study sessions, including fully automated, AI-based data analysis pipelines that were used directly from the rural villages via mobile networks. The lack of group difference is particularly significant given the notably less free playtime recorded in Malawi (supervised recordings) compared to the reference recordings in Finland (unsupervised recordings) (Supplementary file 1). In practice, the findings also support the idea that MAIJU wearable data can be processed with this analysis pipeline without direct monitoring and manual note taking by the on-site study assistants.

The quantified motor assessment with at-home wearable recordings offers several advantages compared to the existing methods: It overcomes the many practical challenges of hospital/lab-based assessments, including their need for trained experts and the unnatural settings from the infants’ perspective. Measuring infants’ spontaneous activity in their natural environment increases the ecological validity of the method. Currently used questionnaire-based methods have attempted to count infants’ motor development as “days of experience”,^22^ but they cannot provide objective and accurate measures of the many essential details of infants’ motor performance, such as times spent in or the variability of postures, overall motor maturity, or the movement patterns. They also struggle with accounting for environmental factors related to each infant.^9^

Many studies have used norms from the Western or high-income countries to assess infants in other environments, ignoring the well-established cross-cultural variations.^22–26^ For example, it is well shown that cultural disparities in mother’s expectations and child-rearing practices result in cross-cultural variance in early (motor) development.^22,27–32^ During this feasibility study, MAIJU wearable recording sessions were accompanied by other children from the neighboring families, whereas infants of the same age in Finland were more likely to be surrounded by toys. Otherwise, the recording environment i.e., recording surface and the building materials of the house did not have a significant effect on the technical data quality of this study. Such differences in the infant’s lived experience are likely to affect motor performance, which can only be studied by using reliable and quantitative methods that assess motor performance at the infant’s own home.

We did not encounter any major limitations or persisting problems in the rural Malawian setting. As the infants often spent their time on the ground or concrete floors, we saw suits’ heavy wear and tear more than was seen earlier in Finland. This can be fixed readily by supporting local textile repair, and by increasing the mechanical durability of the suit material (e.g. using sewn fastening straps instead of glued ones). During the initial stages of the study, some technical challenges were experienced with the Bluetooth connections of a specific iOS device model, which was resolved by switching to new iOS devices. This trouble shooting required some additional training of the data collectors to ensure that the infant remains within Bluetooth range, and to manage possible crashes of the mobile application. All issues with Bluetooth connections will be solved with the ongoing introduction of new sensors that store data on the sensors instead of continuous streaming to the mobile device. That will also support much longer recording times when needed, which can only be studied using reliable and quantitative methods that assess motor performance at the infant’s own home.^33^ Such a phone-free option was not available by the start of the present feasibility study, so it was performed using real-time data streaming.

Finally, an environment like rural Malawi may experience extreme seasonal variations in the weather conditions and food security, which can indirectly affect infants’ motor performance (hunger, fatigued; less active) and pose an external confounder to the results. These effects were not systematically studied, but our data collectors reported that often in the morning hours, the family had not made porridge before the data collector arrived, so the measurement session had to start by feeding the infants with dairy blend juice before starting the recording session. Furthermore, conditions of the roads leading to the households varied significantly. Some areas were accessible via tarmac roads, although these were often marked by frequent potholes. In other cases, dirt roads or narrow paths were the only routes available. Accessibility was sometimes further compromised during the rainy season when heavy rainfall made certain routes impassable. While these road conditions were not directly related to the MAIJU method itself, they occasionally necessitated the rescheduling of appointments to accommodate for travel disruptions.

For a more widespread use of the MAIJU wearable system in environments of this kind, it is important to consider the need for personnel responsible for new study methods, the logistics related to new devices (the suits, sensors, mobile devices, batteries) as well as the mechanisms for local repair after tear and wear. Yet, wearable methods may offer a widely scalable option for a distributed health monitoring, and the novel AI-based algorithms support equality by providing the same analytics to measurements in any parts of the world. As per our previous experience with the clinical trials in Finland, all components in the MAIJU system, apart from the batteries, can be recycled tens or hundreds of times, reducing the overall measurement-wise cost. As a benchmark, the current small series production and the presently used components cause measurement-wise costs at the range of 5-10$. Notably, the costs could go much lower with a more optimized, large volume industrial production, and even at these levels vast majority of the study spendings come from other costs, such as personnel and logistics. At the same time, more local savings come from reducing the need of patient and parent transportation, and a potentially reduced need for expert visits. Ultimately, the key issue en route to a wider uptake is proving a sufficient added value in each use case. Our ongoing work aims to establish motor growth charts from the rural Malawi population, to offer a strong benchmark for future studies in comparable environments. The present findings encourage further work to establish the practical routes to relevant skills transfer to the low resource countries, and the aspects of added value of wearable methods in a range of developmental studies, in health care, and in other global health research. The successful usage of MAIJU wearable in a low-income rural setting is promising for communities and health systems where children’s development assessment skills and resources are scarce. Such AI-based developmental assessment could potentially be an affordable way to diagnose and follow up developmental delays in primary health care and hospitals in resource-limited settings.

## Supplementary information

Supplementary information

## Data Availability

The datasets generated during and/or analyzed during the current study are not publicly available due to ethical restrictions related to the involvement of human participants but are available from the corresponding author on reasonable request.
