# Correction: Assessing motor development with wearables in low-resource settings: feasibility in rural Malawi

**DOI:** 10.1038/s41390-025-04019-8

**Published:** 2025-03-21

**Authors:** Elisa Taylor, Manu Airaksinen, Rikhard Ihamuotila, Milja Kivelä, Ulla Ashorn, Leena M. Haataja, Charles Mangani, Sampsa Vanhatalo

**Affiliations:** 1 BABA Center, Pediatric Research Center, Department of Clinical Neurophysiology, New Children’s Hospital and HUS Imaging, Helsinki University Hospital, Helsinki, Finland; 2 Department of Physiology, University of Helsinki, Helsinki, Finland; 3 Center for Child, Adolescent and Maternal Health Research, Faculty of Medicine and Health Technology, Tampere University and Tampere University Hospital, Tampere, Finland; 4 Research Unit of Clinical Medicine, MRC Oulu, Oulu University Hospital and University of Oulu, Oulu, Finland; 5 Department of Pediatric Neurology, Children’s Hospital, Helsinki University Hospital and University of Helsinki, Helsinki, Finland; 6 Kamuzu University of Health Sciences, Blantyre, Malawi

Correction to: *Pediatric Research* 10.1038/s41390-025-03818-3, published online 03 March 2025

In this article ref. 33 was incorrect and should have been ‘Airaksinen, M. et al. Assessing Infant Gross Motor Performance With an At-Home Wearable. Pediatrics, e2024068647 (2025).’

The original article has been corrected.

