# Supplementary material for: Assessing motor development with wearables in low-resource settings: feasibility in rural Malawi
Source: Pediatr Res. 2025 Mar 3;99(7):2444–50. doi: 10.1038/s41390-025-03818-3 (PMC13485677; doi:10.1038/s41390-025-03818-3)
Supplement: Supplementary file 1 [file 41390_2025_3818_MOESM1_ESM.pdf]

Supplemental file 1

		MALAWI		FINLAND		STATISTICS
**Participants**		44		47		χ^2 = 47.000, df = 46, p = 0.431
**Male/Female**	**Male%/ Female%**	22/22	50%/50%	25/22	53%/47%	χ^2 = 0.010, df = 1, p = 0.919
		**Mean (SD)**	**Median (range)**	**Mean (SD)**	**Median (range)**	
**Age (months)**		7.4 (1.2)	7.0 [5.0-10.0]	7.4 (1.6)	7.4 [4.1-10.0]	t(230) = 0.166, p = 0.868
**Number of recordings**		121		111		χ^2 = 232.000, df = 1, p < 0. 001
**Mean recording time (mins) of the total number of recordings**		M = 99.8 (SD = 33.6)		M = 559.1 (SD = 207.5)		U = 314.000, Z = -12.536, p < 0.001
**Free playtime (min), automated analysis**		M = 38.6 (SD = 31.1)		M = 118.4 (SD = 69.9)		U = 2063.000, Z = -9.121, p < 0.001
**Free playtime (min), manual analysis**		M = 65.1 (SD = 34.9)		M = 236.3 (SD = 118.7)		U = 1275.500, Z = -10.653, p < 0.001

Participant & dataset characteristics: Malawi / Finland.

Supplemental file 2

Family characteristics		
	**N**	**%**
**Number of people in the household**		
3-5	26	60.5
6-7	12	27.9
8-10	5	11.6
**Mother's tribe**		
Yao / Chewa	40 / 3	93.0 / 7.0
**Father's tribe**		
Yao / Chewa / Lomwe	40 / 2 / 1	93.0 / 4.7 / 2.3
**Can the mother read?**		
No / With difficulties / Fluently	28 / 15 / 0	65.1 / 34.9 / 0.0
**Can the mother write?**		
No / With difficulties / Fluently	26 / 17 / 0	60.5 / 39.5 / 0.0
**Can the father read?**		
No / With difficulties / Fluently	14 / 28 / 1	32.6 / 65.1 / 2.3
**Can the father write?**		
No / With difficulties / Fluently	14 / 28 / 1	31.8 / 63.6 / 2.3
**Maternal / paternal occupation**		
Farming	22 / 3	51.2 / 7.0
Fishing	1 / 17	2.3 / 39.5
Stays at home / no job	14 / 1	32.6 / 2.3
Other vending	1 / 5	2.3 / 11.6
Piece worker	4 / 11	9.3 / 25.6
Selling potatoes	1 / -	2.3 / -
Office worker	- / 2	- / 4.7
Tailor / seamstress	- / 2	- / 4.7
Milling work	- / 1	- / 2.3
Motorbike mechanic	- / 1	- / 2.3

Family characteristics and socio-demographic background of infants' developmental environment, Malawi (n = 43)[a].

[a]The total number of participants in the feasibility study was n = 44.

Supplemental file 3

a) Malawi Finland

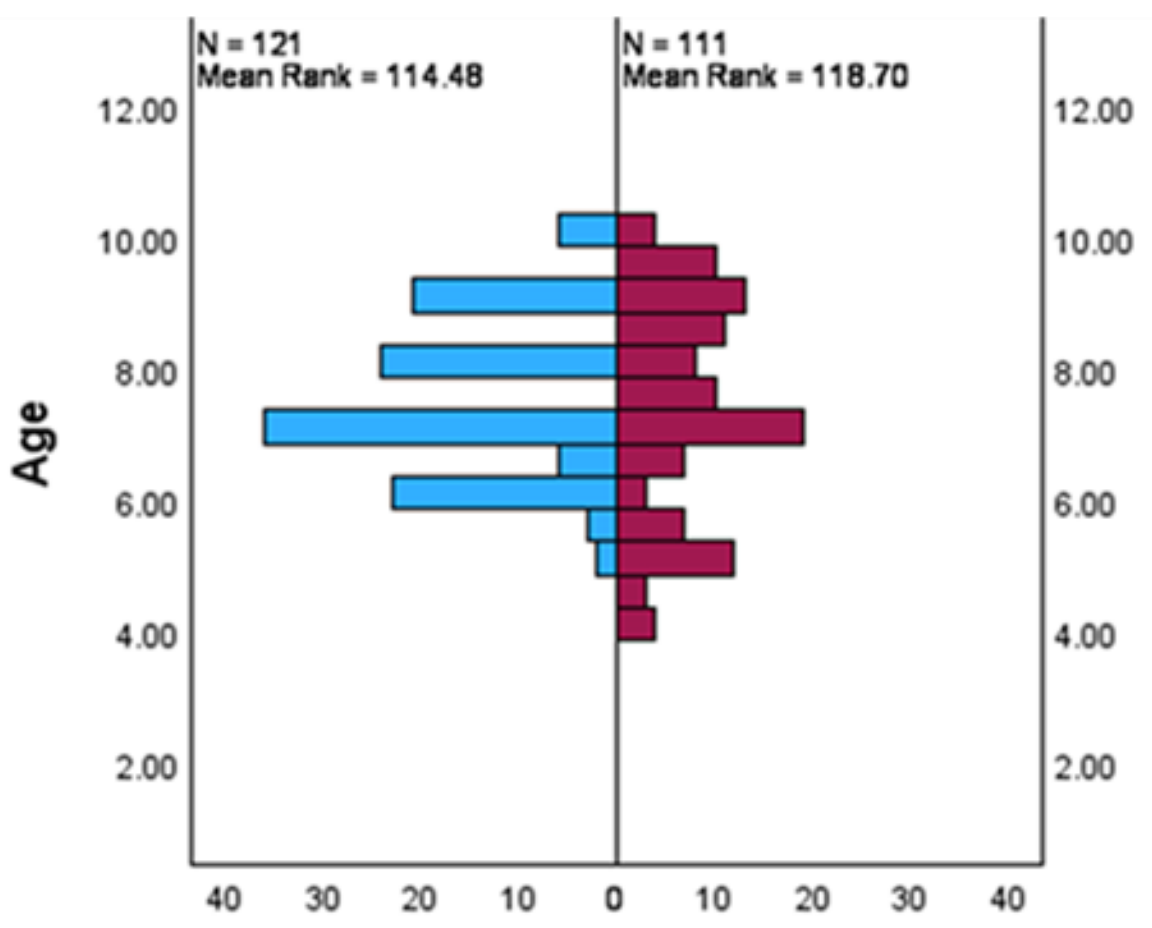

b) Age at failed / successful recordings

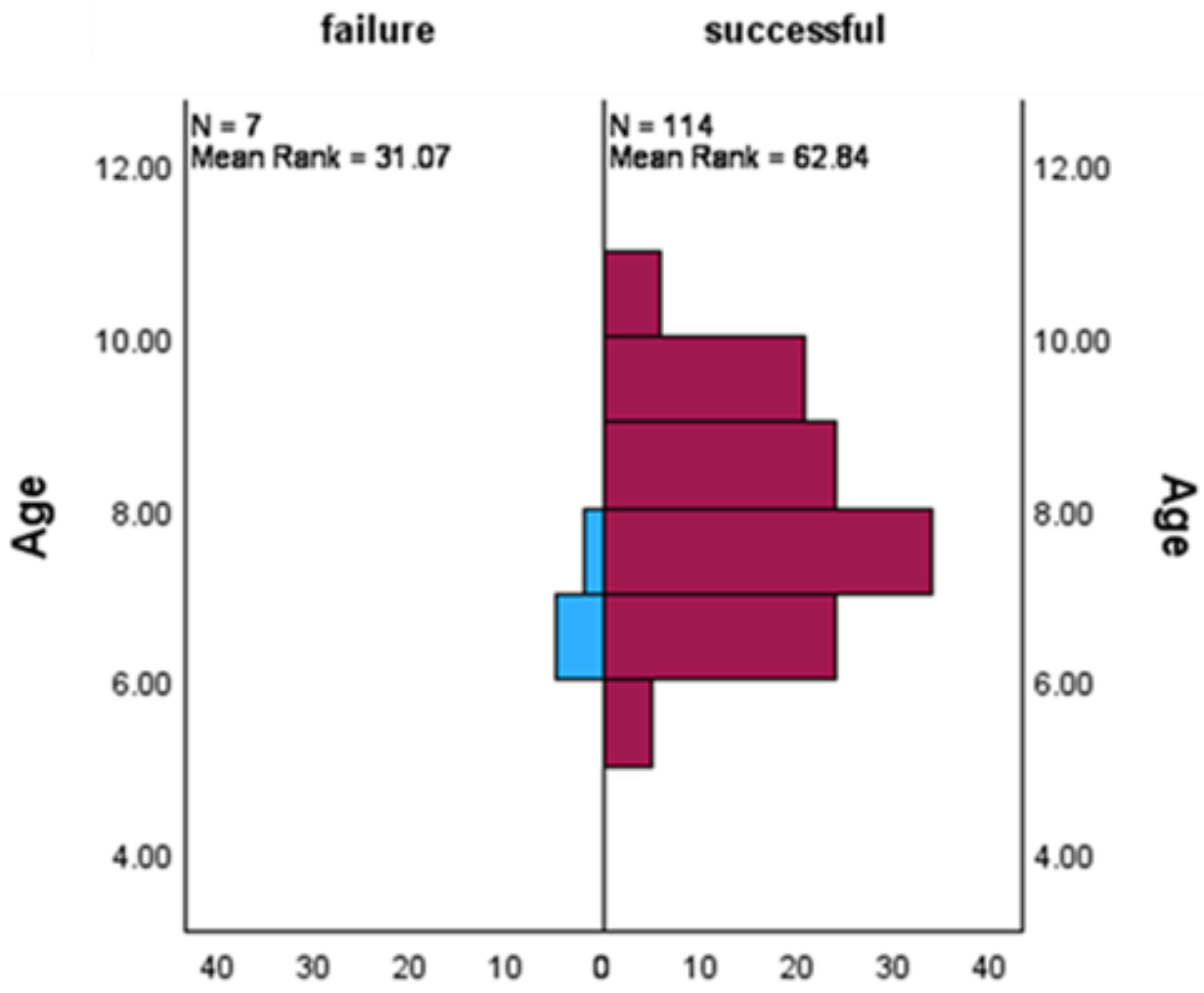

a) Age distribution by recording (Malawi, Finland), and b) Age distribution for failed vs. successful recordings (Malawi).

Supplemental file 4

	**10. User Experience**					
10.1	Did you face any challenges with using the smart jumpsuit			Multiple choice	0, No │ 1, Yes │99, Not known	
10.1.1	If yes, please describe			text		
10.2	Did you see any markings on the skin due to the jumpsuit?			Multiple choice	0, No │ 1, Yes │99, Not known	
10.2.1	If yes, please describe			text		
10.3	How do you find the jumpsuit?			Multiple choice	0, Very bad │ 1, Reasonable │2, Good │3, Excellent	
10.3.1	Do you want to describe?			text		
10.4	Was your child bothered about the jumpsuit?			Multiple choice	0, No │ 1, Yes │99, Not known	
10.4.1	If yes, please describe			text		
10.5	Did the jumpsuit affect your child's behaviour?			Multiple choice	0, No │ 1, Yes │99, Not known	
10.5.1	If yes, please describe			text		
10.6	Would you like to change the jumpsuit somehow?			text		

Maternal User Experience Questionnaire (Electronic Data Collection Form)
